# Action of Antiseptics on Toxin of Bacillus Welchii

**Published:** 1918-12

**Authors:** 


### ABSTRACTS

### SURGERY

***The Action of Antiseptics on the Toxin of Bacillus Welchii. A Preliminary Note.*** By HERBERT D. Taylor, M. D., and J. HAROLD AUSTIN, M. D. *The Journal of Experimental Medicine.* March 1, 1918.

The destructive action of the antiseptics in general use on bacterial toxins has been made conspicuous first by Carrel and Dehelly, secondly by Lumière.

Carrel and Dehelly considered that the improvement of the general symptoms in patients with infected wounds treated with hypochlorite was due to a reduction in the amount of toxin absorbed; whereas Lumière had found that pus containing virulent organisms together with bacterial toxins became inocuous after addition of hypochlorite.

As a sequel to these observations, the authors decided to try to measure quantitatively the action of antiseptics on a special toxin widely met with in military surgery.

Experiments had to be undertaken with a definite toxin which could be measured, and a suitable animal as an indicator. Both these conditions were realized by undertaking the tests on pigeons with the toxin of Bacillus Welchii, Bull and Pritchett having previously standardized the virulence of that toxin.

*Method. Production of Toxin.* Virulent strains of B. Welchii are incubated for 18 hours in an adequate culture medium. Then the fluid is centrifuged for 20 minutes at high speed, and filtrated through a Berkefeld N candle. The different lots of toxin produced in this way were found to differ considerably in virulence.

In all cases the toxic filtrate was titrated previously to its use. The smallest amount which would kill a pigeon weighing from 300 to 400 gr. in 12 hours or less was determined and considered as one lethal dose.

*Treatment of Toxin with Antiseptic.* Volumes of filtrate containing the required number of fatal doses were measured. Horse serum, inactivated at 58°C. for an hour was added to the solutions in some experiments. Sodium chloride solution at 0.9 per cent.

was added to the portions requiring additional volume, and the antiseptic was added last of all. The whole remained for five minutes in contact and then was injected into the pectoral muscles of a pigeon.

The results of one experiment are summarized below in a tabular form.

| Fatal doses of toxin. | ANTISEPTIC | Horse serum. | Na Cl solution. | RESULT. |
|---|---|---|---|---|
| 1 | . . . . . . . . . . . . . . . . . . | | 7 | Died in 12 hours. |
| 3 | 5 c.c. of Dakin's solution . . | | | Lived. |
| 6 | 2 " " " " . . | | | " |
| 6 | 2 " " " " . . | 4 | | " |
| 3 | 5 c.c. of phenol solution . . | | | Died in 5 hours. |
| 3 | 5 c.c. of chloramine. T. sol.. | | | Lived. |
| 3 | 5 " " " " .. | 4 | | " |
| | 5 c.c. of Dakin's sol. . . . . | | 3 | " |
| | 5 c.c. of phenol. . . . . . . | | 3 | " |
| | 5 c.c. of chloramine. T. sol.. | | | " |

1 fatal dose of toxin killed the pigeon in test N° 1; whereas 2 cc. of Dakin's sol. protected the pigeon against 6 fatal doses. 2 cc. of Dakin's sol. also proved effective enough, even in the presence of 4 cc. of horse serum, although blood serum causes hypochlorite sol. to decompose and reduces the efficiency of that antiseptic.

Phenol does not show any protective property, whereas chloramine-T sol. proves similar to Dakin's sol.

Control injections of the antiseptics only were performed, and demonstrated that the doses of antiseptics injected were not of themselves toxic.

The authors considered it more precise to make the mixtures of toxin and antiseptic *in vitro*. But it may well be supposed that these solutions exert a similar influence when used in the treatment of wounds. The fact that the detoxicating action was still demonstrable, when the toxin was treated with serum before the addition of the antiseptic, gives to these results great practical importance. The conditions in the last case closely simulate those encountered when the antiseptic is applied to wounds.

On the whole Dakin's sol. and chloramine-T sol. have a marked detoxicating power against the toxin of B. Welchii. Phenol sol., 0.25 per cent., has no such action.

The observations are not recorded with the purpose of advocating the use of an antiseptic in the place of specific antitoxins

In human surgery, the antiseptic treatment of infected wounds will doubtless be combined with specific serum therapy.

